# FGFR作为肺鳞癌潜在治疗靶点的研究进展

**DOI:** 10.3779/j.issn.1009-3419.2018.02.05

**Published:** 2018-02-20

**Authors:** 明 董, 彤 李, 军 陈

**Affiliations:** 300052 天津, 天津医科大学总医院肺部肿瘤外科 Department of Lung Cancer Surgery, Tianjin Medical University General Hospital, Tianjin 300052, China

**Keywords:** FGFR, 肺鳞癌, 治疗靶点, FGFR, SqCLC, Targeting

## Abstract

肺鳞状细胞癌（squamous-cell lung cancer, SqCLC）是非小细胞肺癌中一类独特的病理类型，患者多为高龄、发病隐匿、发现时常属晚期、常伴有心肺合并症、缺乏有效的靶向治疗药物等因素，相对于非鳞非小细胞肺癌，SqCLC的治疗面临着更大的挑战。近年针对肺癌的分子靶向药物迅速发展，我们发现，FGFR家族（FGFR1-4）基因改变存在于约12%的SqCLC中，是SqCLC中突变频率最高的酪氨酸激酶家族基因，同时许多靶向FGFR的小分子药物都在各类肿瘤中发挥了较好的治疗效果。目前，许多FGFR抑制治疗SqCLC的临床试验也都正在进行当中，可能为SqCLC治疗提供新的策略和方向。

## Abstract (en)

Squamous cell lung cancer (SqCLC) is a unique clinical and histologic category of non-small cell lung cancer (NSCLC). Most of patients with SqCLC tend to be older, typically at advanced stage, associated with smoking and have more complications. With progress of targeted therapy of lung cancer, we identified several potential actionable genetic abnormalities such as FGFR. Several FGFR inhibitors have been approved for clinical use in different cancers. And some of these agents are currently under investigation in clinical trials for SqCLC. This article summarizes the current knowledge about FGFR aberrations, the relative inhibitors in development and clinical data in SqCLC.

肺鳞状细胞癌（squamous-cell lung cancer, SqCLC）是非小细胞肺癌中一类独特的病理类型，约占非小细胞肺癌总数的25%-30%^[1]^。临床上，SqCLC多为中心型肺癌，沿近端支气管生长，常侵犯大血管^[2]^。由于其独特的病理特征^[3]^、患者多为高龄^[4]^、发病隐匿、发现时常属晚期、常伴有心肺合并症^[5]^、缺乏有效的靶向治疗药物等^[6]^因素，相对于非鳞非小细胞肺癌，SqCLC的治疗面临着更大的挑战。近年针对肺腺癌的分子靶向药物迅速发展，极大的改善了患者的预后，然而，SqCLC尚无安全有效的分子靶向药物应用于临床。随着基因测序技术的发展应用，研究者们发现了一些针对SqCLC的有潜在治疗价值的基因突变，例如SOX2扩增、TP63的扩增和过表达、*NFE2L2*和*KEAP1*突变、PI3K信号通路的改变、FGFR1扩增以及*DDR2*突变^[7]^。除此之外，肿瘤基因图谱计划（The Cancer Genome Atlas, TCGA）的数据显示*CDKN2A*、*MLL2*、*NOTCH1*、*RB1*和*HLA-A*基因突变也广泛存在于SqCLC中。结合多方面的数据发现，FGFR家族（FGFR 1-4）基因改变存在于约12%的SqCLC中，是SqCLC中突变频率最高的酪氨酸激酶家族基因^[8]^。而目前关于FGFR作为SqCLC治疗靶点的研究也取得了一定进展，本文对此做一综述。

## 1 纤维母细胞生长因子受体和配体

纤维母细胞生长因子受体（fibroblast growth factor receptor, FGFR）的信号通路在肿瘤生长的过程中发挥了巨大作用，因此其成为多种肿瘤的潜在治疗靶点。FGFR家族包含4个成员：FGFR 1-4，分别由不同的基因编码，均属于受体酪氨酸激酶，包含一个细胞外的配体结合位点、转膜结构域和细胞内活化激酶的催化结构域^[9]^。*FGFR*的基因异常扩增及各组突变多见于肺鳞癌、子宫癌和膀胱癌^[10]^。当配体成纤维细胞生长因子（fibroblast growth factor, FGF）与FGFR结合时，诱导受体二聚化，导致酪氨酸激酶细胞内结构域转磷酸化，激活下游信号通路。目前已经发现人类有22种FGFs，包含与受体结合所必须的高度同源性序列。此外，大部分FGFs包含硫酸肝素蛋白多糖（heparin sulfate proteoglycan, HSPG）区。FGFs与HSPG结合，保护配体不被降解，同时也参与到形成FGFs/FGFRs复合体的过程当中^[11]^。FGFs/FGFRs的主要的下游信号通路包括RAS/MAPK和PI3K/AKT/mTOR^[12]^。因此，FGFR信号通路在细胞增殖、分化、血管生成、损伤修复中发挥着重要的作用。而FGF1和FGF2则与血管内皮生长因子（vascular endothelial growth factor, VEGF）以及血小板源生长因子（platelet-derived growth factor, PDGF）协同作用，促进血管生成^[13]^。FGFs和FGFRs在许多上皮和间质来源的细胞核组织中都有表达，并且在胚胎发育期参与不同器官的生长、分化及血管生成。目前已知特定FGFR2和FGFR3的点突变与先天性骨骼疾病有关^[14]^。成年以后，FGF/FGFR在炎性反应以及组织修复中血管生成和成熟的过程中发挥重要的作用^[12, 13]^。

## 2 肺鳞癌相关的*FGFR*突变

有报道^[8]^显示，在SqCLC样本中，检测到8号染色体短臂12区的基因扩增，而这部分基因包含了*FGFR1*。有研究^[8]^通过荧光原位杂交（fluorescence *in situ* hybridization, FISH）的方式证实，在153例SqCLC样本中，约有22%样本存在FGFR1的扩增。而Hammerman等^[15]^通过通过单核苷酸多态性芯片（single nucleotide polymorphism, SNP）分析显示，约有21% SqCLC样本存在8号染色体短臂11-12区域基因扩增，同时证实了存在FGFR1扩增的非小细胞肺癌系细胞生长取决于FGFR1的激活。同时，吸烟人群FGFR1扩增概率高于不吸烟人群^[16]^。除此之外，尚未发现其他临床特征与FGFR1相关。Kim等^[17]^的研究显示，手术切除且伴随FGFR1扩增的SqCLC患者，无病生存期（diseases-free survival, DFS）以及总生存期（overall survival, OS）明显低于不伴有FGFR1扩增的患者（26.9个月*vs* 94.6个月，*P* < 0.001；51.2个月*vs* 115.0个月，*P*=0.002）。多因素分析^[18]^也显示，*FGFR1*基因扩增是预后不佳的独立因素。一项纳入13项研究、包含1, 798例研究对象的*meta*分析显示，SqCLC中FGFR1扩增率为19%，与吸烟及淋巴结转移明显相关，与性别、种族、分期、检测方式以及OS不相关^[16]^。进一步研究显示，*FGFR1*基因扩增的异质性会影响其对FGFR抑制剂的敏感性，而当同时伴有染色体8短臂12区（包括FGFR1）扩增以及染色体11长臂13区（包括CCND1、FGF4及FGF19）扩增时，对FGFR抑制剂则更加敏感。因此，这些证据提示FGFR1抑制剂可能使特定患者受益^[19]^。

在SqCLC病例中，*FGFR*突变可能发生于4种亚型的任何一种，其中FGFR2和FGFR3发生率较高^[8]^。最新的TCGA数据库的信息显示，3%的样本存在FGFR2和FGFR3至少其中一种的基因突变。这些突变多为基因的细胞外结构域中FGFR2（W290C和S320C）、FGFR3（R248C和S249C）以及激酶结构域中FGFR2（K660E和K660N）^[20]^。*FGFR2*和*FGFR3*基因突变在子宫内膜癌^[21]^、宫颈癌^[22]^和膀胱癌^[23]^中也广泛存在。例如FGFR2（K660E和K660N）以及FGFR3（R248C和S249C），这些基因驱动其他肿瘤的生长，同时也暗示这些基因可能也是SqCLC的驱动基因^[24]^。这些研究成果为FGFR和多激酶抑制的潜在治疗作用及靶点提供了理论基础^[20]^。

*FGFR1/3*基因融合在非小细胞肺癌中发生的概率约为1%^[25]^，在SqCLC中发生的概率约为2%-3.5%^[26]^。染色体重排导致了FGFR激酶结构域融合蛋白的表达。其中，FGFR3融合酸性卷曲转化相关蛋白3（transforming acidic coiled-coil containing protein 3, TACC3）在许多肿瘤中都有表达。韩国对104例肺鳞癌患者进行全基因组测序，分析发现1.92%（2/104）样本显示出FGFR3/TACC3融合^[27]^。在TCGA的数据库种，有2.24%（4/178）样本存在FGFR3/TACC3融合^[28]^，这种融合导致FGFR3活性的增加和失调，继而影响其下游信号通路，这也进一步证明FGFR-TACC3融合蛋白具有显著的致癌作用^[28]^。

除此之外，目前认为一些遗传变异，如种系单核苷酸多态性（single nucleotide polymorphisms, SNP），会增加肿瘤的风险。有研究证实，*FGFR2*基因的特定变异会增加乳腺癌的发病率^[29]^；而*FGFR4*基因多态性（Gly338Arg）则与包括肺腺癌在内的不同类型肿瘤的进展相关^[30, 31]^。数据显示，Gly388Arg多态性与肺腺癌患病年龄（*P*=0.002）、分期（*P*=0.002）及不良预后（*P*=0.007）显著相关^[31]^。一项基于RNA干扰技术的小鼠肿瘤基因检测结果显示，24种肿瘤抑制基因（tumor suppressor genes, TSGs）在SqCLC样本中明显下调，部分TSGs可以抑制FGFR信号通路，相应地下调这些TSGs则可以激活FGFR信号通路。这些数据也暗示，FGFR信号通路的变异可能在没有基因扩增、融合或突变的情况下驱动SqCLC癌基因激活^[32]^。

## 3 针对肺鳞癌*FGFR*基因靶向药物的应用与研究进展

AZD4547是一类选择性FGFR1-3抑制剂，在动物实验表现出较强的肿瘤抑制作用，在体内及体外实验中，均显示出对FGFR1扩增驱动的SqCLC的抑制作用^[33]^。在临床试验方面，一项Ib期临床试验显示15例Ⅳ期SqCLC患者经AZD4537（80 mg，口服，2次/日）治疗后，最常见的副反应为胃肠道反应或者皮疹，3例患者由于副反应停药，1例患者部分缓解（complete response, PR），4例疾病稳定（stable disease, SD）^[34]^。一些临床试验子研究的数据显示，AZD4548对于存在*FGFR*扩增的SqCLC患者显示出较好的耐受性，但是治疗作用有限。

JNJ-42756493是一类针对FGFR1-4的多靶点酪氨酸激酶抑制剂，在肺、肝及肾脏组织细胞系中可以靶向抑制FGFR1-4信号通路^[35]^。目前，Ⅰ期临床试验和Ⅱ期剂量爬坡试验显示该药物的推荐剂量为9 mg/d。针对*FGFR*扩增及异位融合的肺癌和乳腺癌二期临床试验也正在进行^[36]^。同时，JNJ-42756493也在进行针对亚裔人群恶性肿瘤的Ⅱ期临床试验。

GSK3052230也被称为FP-1039，是一类FGF配体抗体型药物，可以结合所有与有丝分裂相关的FGF配体，抑制FGF促进的细胞增殖以及FGF（VEGF）诱导的血管生成，从而抑制肿瘤生长。同时GSK3052230不与激素相关的FGF配体（FGF19、FGF21和FGF23）相结合，避免了抑制FGF23引起的高磷血症。一些临床前研究显示，GSK3052230对存在FGFR信号通路变异的许多类型肿瘤都表现出了很强的抗肿瘤作用，特别是针对FGFR1扩增的肺癌以及*FGFR2*突变的子宫内膜癌^[37]^。目前，一项关于GSK3052230安全性与有效性的IB期临床试验正在进行当中，这其中包括3个亚组：A组为GSK3052230联合紫杉醇+卡铂，用于初治的晚期SqCLC；B组为联合多西他赛，用于FGFR1扩增的转移性SqCLC二线治疗；C组为联合培美曲塞+顺铂，用于恶性胸膜间皮瘤的治疗。这项研究主要用于评估GSK3052230安全性和有效性，目前已经结束入组，其结果也是值得期待的^[38]^。

尼达尼布（nintedanib, BIBF1120）是一类针对VEGFR1-3、PDFGRA-B以及FGFR1-4的多靶点血管激酶抑制剂，在细胞水平和动物模型上显示出抑制肿瘤生长的作用^[39]^。Ⅰ期临床试验显示包括非小细胞肺癌在内的多种实体瘤患者对其具有良好耐受性，该药可用于单药或联合化疗，主要副反应包括胃肠道反应、肝酶升高以及乏力。目前已经开展了2项针对尼达尼布治疗过的晚期非小细胞肺癌的多中心随机对照Ⅲ期临床试验（LUME-lung1和LUME-lung2）。LUME-lung1入组1, 314例非小细胞肺癌患者，尼达尼布（*n*=655）及安慰剂（*n*=659）联合多西他赛，结果显示，无论是SqCLC还是肺腺癌组，尼达尼布都明显延长了PFS（3.4个月*vs* 2.7个月，HR=0.79，*P*=0.001, 9），二期尼达尼布还提高了肺腺癌的OS（12.6个月*vs* 10.3个月，HR=0.83，*P*=0.035, 9），但是没有延长SqCLC的OS（HR=1.01, *P*=0.890, 7），主要的不良反应是腹泻和肝酶升高^[40]^。目前还有一些尼达尼布单药或者联合化疗治疗非小细胞肺癌包括SqCLC的Ⅰ期/Ⅱ期临床试验正在进行。

## 4 存在的问题与展望

目前，多种选择性或者多靶点的FGFR抑制剂均表现出对包括SqCLC在内的NSCLC的治疗作用。而现有的临床资料还不是很成熟，尽管针对肺腺癌的部分药物已经进入临床应用，但大部分针对SqCLC的FGFR抑制剂还处于Ⅱ期或Ⅲ期临床试验的阶段。未来也会开展FGFR抑制剂联合EGFR-TKI药物治疗的临床试验，使得EGFR-TKI耐药后的治疗更加丰富灵活。

FGF/FGFR信号通路的突变具有重要的临床意义，特别是对SqCLC。而在多种*FGFR*突变中，FGFR1扩增在SqCLC中发生率最高。现有的研究资料也显示，FGFR信号通路突变可能成为预后不佳的指标，同时也是FGFR抑制剂的潜在干预靶点。随着二代测序技术的发展，基因检测也成为指导个体化治疗重要的手段，这让FGFR抑制剂的应用变得更有针对性。目前，许多FGFR抑制剂相关临床试验都正在进行当中，我们也希望这些试验的结果能给SqCLC的治疗带来新的曙光。
